# Supplementary figures and images for: Transfer of microbiota from lean donors in combination with prebiotics prevents excessive weight gain and improves gut-brain vagal signaling in obese rats
Source: Gut Microbes. 2024 Nov 1;16(1):2421581. doi: 10.1080/19490976.2024.2421581 (PMC11540078; doi:10.1080/19490976.2024.2421581)

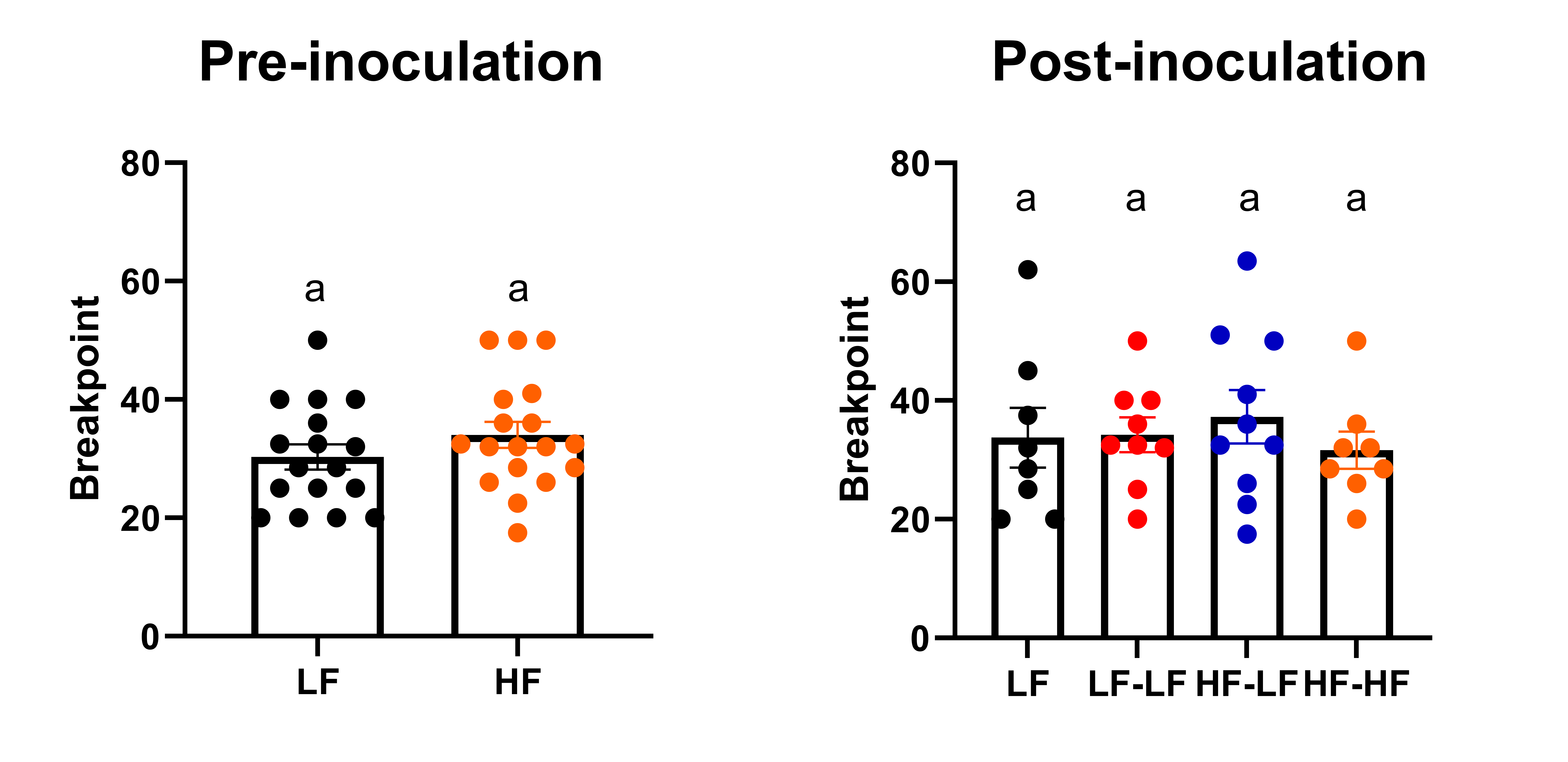

Supplement: Supplemental Material [file KGMI_A_2421581_SM8830.zip › Supplementary material-KGMI_A_2421581/kgmi-s-2024-1123-20241022202803/New SuppFig4.tif]

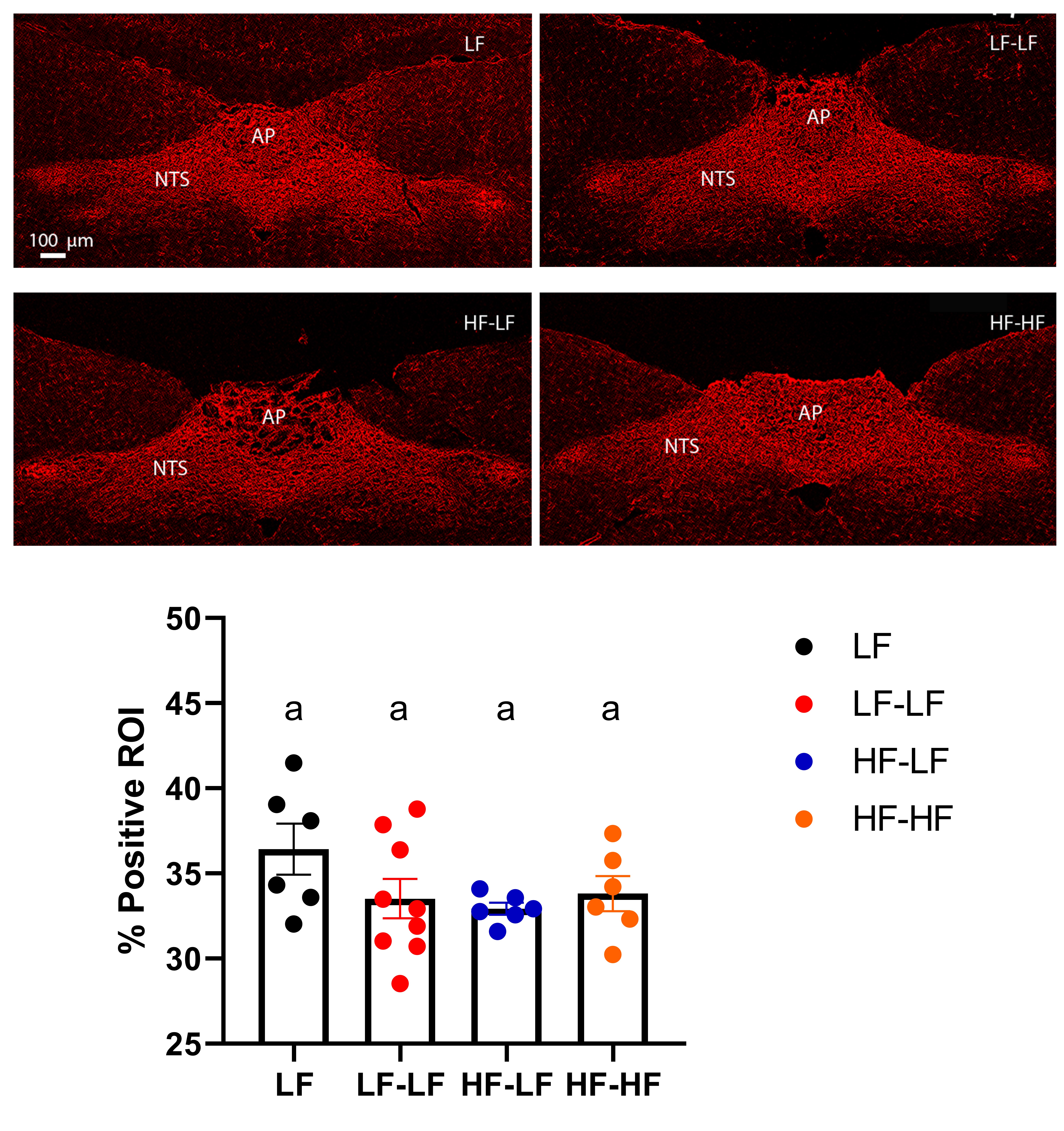

Supplement: Supplemental Material [file KGMI_A_2421581_SM8830.zip › Supplementary material-KGMI_A_2421581/kgmi-s-2024-1123-20241022202803/New SuppFig5.jpg]

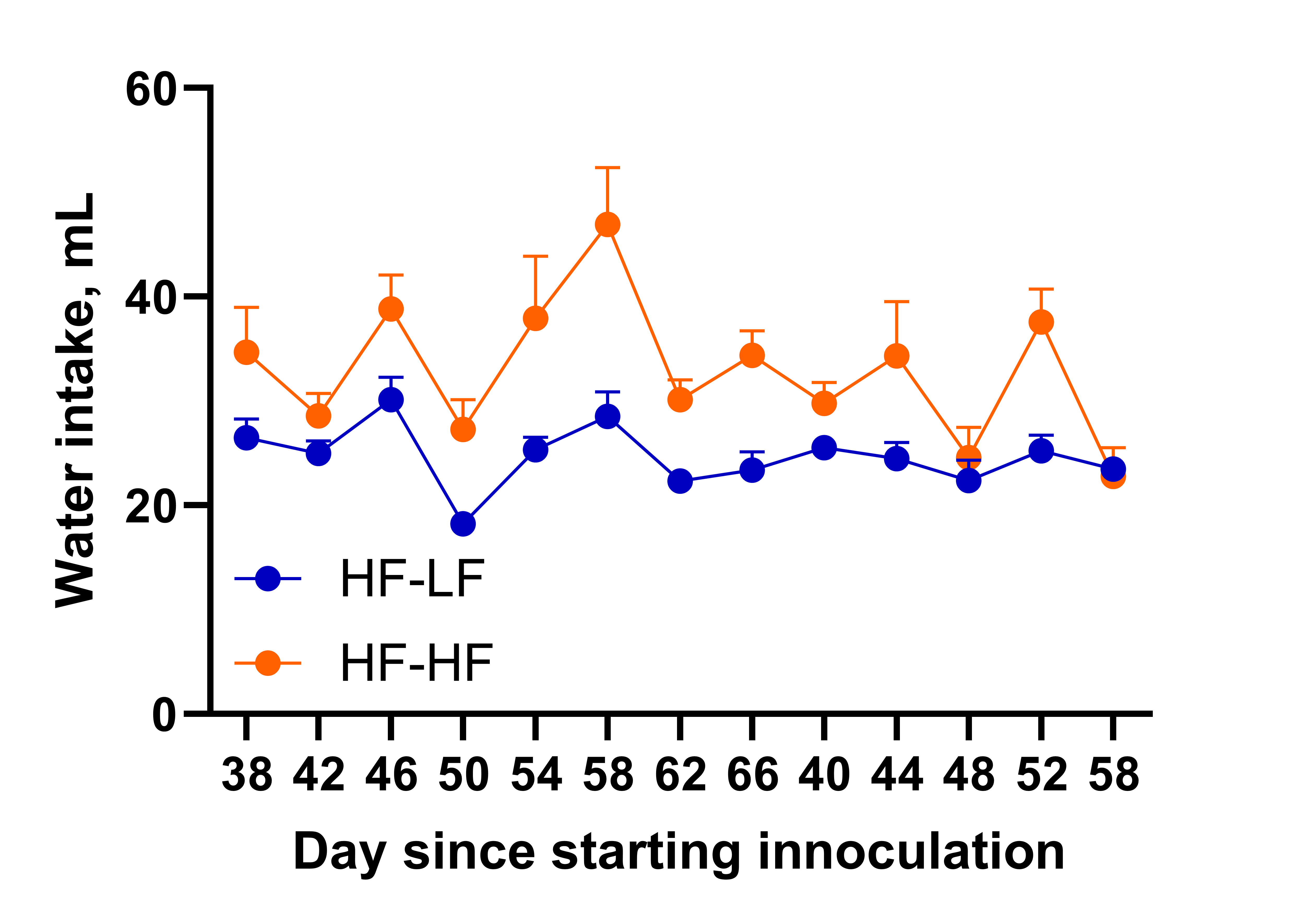

Supplement: Supplemental Material [file KGMI_A_2421581_SM8830.zip › Supplementary material-KGMI_A_2421581/kgmi-s-2024-1123-20241022202803/SuppFig1.tif]
